# In Vivo Effects of *Crataegus pinnatifida* Extract for Healthy Longevity

**DOI:** 10.4014/jmb.2302.02022

**Published:** 2023-03-10

**Authors:** In-sun Yu, Mina K. Kim, Min Jung Kim, Jaewon Shim

**Affiliations:** 1 Division of Food Functionality Research, Korea Food Research Institute, Wanju 55365, Republic of Korea; 2 Department of Food Science and Human Nutrition and K-Food Research Center, Jeonbuk National University, Jeonju 54986, Republic of Korea; 3 Department of Biochemistry, Kosin University College of Medicine, Busan 49267, Republic of Korea

**Keywords:** Healthy longevity, *Crataegus pinnatifida*, nutrient-sensing pathway, drosophila melanogaster, oxidative stress resistance

## Abstract

Aging is a complex series of multi-organ processes that occur in various organisms. As such, an in vivo study using an animal model of aging is necessary to define its exact mechanisms and identify anti-aging substances. Using *Drosophila* as an in vivo model system, we identified *Crataegus pinnatifida* extract (CPE) as a novel anti-aging substance. Regardless of sex, *Drosophila* treated with CPE showed a significantly increased lifespan compared to those without CPE. In this study, we also evaluated the involvement of CPE in aging-related biochemical pathways, including TOR, stem cell generation, and antioxidative effects, and found that the representative genes of each pathway were induced by CPE administration. CPE administration did not result in significant differences in fecundity, locomotion, feeding amount, or TAG level. These conclusions suggest that CPE is a good candidate as an anti-aging food substance capable of promoting a healthy lifespan.

## Introduction

Over the past few decades, human life expectancy has steadily increased with the improvements in medical treatment and health care. Although this increase in lifespan is a blessing to humanity, it is associated with various social problems, such as a decrease in the working-age population, and the cost of medical support for the elderly is gradually increasing [1]. Therefore, the ultimate goal is not simply to extend lifespan, but to ensure a healthy lifespan free of long-term disease [2].

Aging is a time-dependent, irreversible, universal occurrence caused by a variety of factors that inevitably culminate in dysfunction [3]. As aging progresses, genetic damage is caused by external factors, including reactive oxygen species (ROS) and physical stimuli [3]. At the molecular level, if damage is not repaired and accumulates, the range of damage gradually expands, resulting in systemic levels of damage that eventually affect lifespan [4]. In other words, aging is accompanied by changes and damage at the systemic level; therefore, it is crucial that aging experiments be conducted in vivo. *Drosophila* species are widely used as an in vivo animal model in aging experiments. *Drosophila* has a shorter lifespan than other model organisms and can be used to obtain results at a low cost and within a relatively short time. The genetic homology of *Drosophila* with humans is very high, and 75%of the genes associated with human diseases are conserved in this model organism [5]. In this study, we used *D. melanogaster* in aging experiments to identify anti-aging substances.

Among the determinants of a healthy lifespan, nutrition is known to affect aging. To determine the effects of dietary intake on lifespan using *Drosophila*, a variety of foods have been studied, including antioxidant-rich foods, such as blueberries [6], cocoa [7], and cranberries [8]. Many previous studies have investigated the correlation between antioxidants and lifespan extension [9, 10]. Several studies are currently being conducted to identify novel anti-aging agents. Here, we tested *Crataegus pinnatifida* extract (CPE) to determine its influence on lifespan. *C. pinnatifida* is used in traditional Chinese medicine as an herb and can be consumed in the form of juices, drinks, jams, and canned fruits [11]. CPE has been associated with a variety of physiological activities, including cardiovascular, digestive, antibacterial, anticancer, immune, osteoporosis, and antioxidant activities. It is composed of over 150 compounds, including flavonoids and steroids [12]. Many previous studies have investigated the efficacy of CPE on physiological activity [13-15]. Despite these studies, research on the effect of CPE intake on lifespan is lacking. In this in vivo study, we sought to confirm that the ingestion of CPE affects not only physiological activity, but also lifespan. The altered expression levels of genes involved in protein synthesis and proteolysis, stem cell generation, and stress resistance influence the extension of lifespan. We measured the expression levels of 4EBP, which is known to be an aging marker by affecting translation and mitochondria respiration, as well as the expression levels of *Atg5* and *Atg8a*, which are known to affect membrane expansion and autophagy completion. We confirmed the expression of genes acting on the Notch pathway and telomeres, both of which are involved in stem cell differentiation [16, 17]. The JNK pathway, which affects stress resistance and lifespan, is regulated by Hep (hemipterous); therefore, we investigated whether CPE affected these pathways [18]. Finally, we attempted to determine the changes in the transcription and expression of genes related to oxidative stress in a control di*et al*one and a diet supplemented with CPE.

## Materials and Methods

### Fly Strain

The *w^1118^* *Drosophila* strain was used as the wild-type *D. melanogaster*. *w^1118^* was obtained from the Bloomington *Drosophila* Stock Center (Department of Biology, Indiana University, Bloomington, IN, USA). Flies were maintained on standard cornmeal-yeast-agar medium (SM) in cages, with 12 h light/12 h dark cycles at 25°C and 60% humidity.

### Fly Food

The pulp of *Crataegus pinnatifida* was purchased from the Korea Plant Extract Bank (KPEB, Korea). Extraction was performed with 50% ethanol for 4 h at 60°C as previously described. The ratio of sample and solvent was 1:20 (w/v). Filtration was performed using 110 nm filter paper (No. 2, Advantec Co., Japan) and concentration using a vacuum concentrator (Eyela New Rotary Vacuum Evaporator, Rikakikai Co., Japan). This samples were kept in a vacuum freeze dryer (Eyela FD1, Rikakikai Co.) and the yield of dried sample was measured. The final products were stored at -20°C before use.

The control diet was made with cornmeal, agar, dextrose, dry yeast, tegosept, and propionic acid. These materials were purchased from Hansol Tech (Korea). This control diet is referred to hereafter as the standard cornmeal-yeast-agar medium (SM). The experimental diet was prepared by adding *Crataegus pinnatifida* extract (SM+CPE) to standard cornmeal-yeast-agar medium. CPE concentration was 1 mg/ml into SM.

### Measurement of Lifespan in *D. melanogaster*

The lifespan assay method used was referred to in the previous study [1]. For preparation of synchronized flies, flies were kept in a cage for 24 h. The collected embryos were washed and collected with 1x phosphate-buffered saline (10x PBS, Welgene, Korea). This method was also conducted referring to the previous paper [2]. The collected embryos were seeded in the food bottle. The 1x PBS concentration containing embryos was 0.1 g/ml. Adult flies were divided into 3 vials (50 flies per vial) by gender. To stabilize them, the flies were maintained in standard medium, and then transferred to vials with or without CPE respectively. These flies were transferred to new food every 2–3 days until every fly died. During transfer, all dead flies were counted and removed.

### Western Blot Analysis

Flies were homogenized in a 1x RIPA buffer (RIPA Buffer, Biosesang, Inc., Korea). Then, proteins were obtained and separated by SDS-PAGE gel. Following that, the gel was transferred to PVDF membranes (Trans-Blot Turbo Mini PVDF Transfer Packs, USA) and blocked using 5% BSA (bovine serum albumin, Sigma, USA) in TBST buffer (10 mM Tris–HCl containing 150 mM NaCl and 0.05% Tween 20, 10x TBST, Biosesang, Korea). Primary antibodies used overnight at 4°C were as follows: SOD1 (Anti-Superoxide Dismutase 1 Antibody, Abcam, UK), SOD2 (Anti-SOD2/MnSOD Antibody, Abcam), catalase (Anti-Catalase Antibody, Abcam, UK), 4E-BP (4E-BP1 Antibody, Cell Signaling Technology, USA), and α-tubulin (Anti-α-Tubulin, Sigma Aldrich, USA). After this process, membranes were thoroughly washed in TBST, and applied with secondary antibodies (Sigma Aldrich, USA). After washing in TBST thoroughly once more, the membranes were detected using a chemiluminescence detection kit. (Thermo Fisher Scientific, USA).

### Real-Time PCR Analysis

Total RNA was extracted from the bodies of whole flies. Flies were homogenized with 10 μl/fly TRIzol (Invitrogen, USA). Extraction was performed according to the TRIzol manufacturer’s protocol. To synthesize cDNA, 1 μl RNA and Superscript III (SuperScript III Reverse Transcriptase, Invitrogen, USA) were used according to the Superscript III manufacturer’s protocol. For cDNA synthesis, real-time PCR was performed by reacting SYBR Green (SYBR Green PCR Master Mix, Applied Biosystems, UK), primer 5 pmole of test gene (Table 1), 1 μl of synthesized cDNA, and DW. The RT-PCR was carried out using on an ABI Prism One-Step Sequence Detection System (Applied Biosystems). The relative amount of target gene was calculated using the 2(Ct^test gene^ – Ct^*Rp49*^) with *Rp49* as an internal control.

### Feeding Assay

Three-day-old flies were divided into male and female, respectively, under CO_2_ anesthesia and stabilized in SM for 24 h. To starve fruit flies, 1% agarose was poured into vials and solidified to make a solid medium. The flies were starved in solid medium with adequate moisture for 15 h. After that, the fruit flies were transferred to vials with reagent composed of 5 mM sucrose and 0.125 mg/ml of Brilliant Blue FCF (Wako Pure Chemical Industries, Japan) in 1% agarose (Certified Molecular Biology Agarose, Bio-Rad, USA) and stored for 90 min. The fruit flies were then homogenized well with 1x PBS, and centrifuged at 12,000 g and 4°C. The absorbance was measured at OD625 nm using a fluorescence spectrometer (SpectraMax M2, Molecular Devices, USA).

### Behavior Test

Measurement of locomotor function was performed as described previously [3]. The flies were fed SM+CPE or SM for 2 weeks and divided by gender. The flies were stocked 30 per each empty vial. By tapping the vials, the flies were made to go down to the bottom. Then, we measured the time it took for 25 flies to climb up to 8 cm vertically.

### Fecundity Assay

For the fecundity assay, a similar protocol that has been described previously was used [4]. We prepared vials that contained apple juice agar plates with SM+CPE or SM for food. One female fly and two male flies were maintained in each of the vials. During 24 h, flies laying embryos were collected at the same time every 10 days with the flies that were fed SM+CPE (1 mg/ml) or SM for 2 weeks. Then, we counted the number of fly embryos that were laid on the apple juice plate surface per vial.

### Hydrogen Peroxide (H_2_O_2_) Test

This experiment was conducted in reference to the previous paper [5, 6]. First, we prepared *Drosophila* that were either fed or went without CPE for 14 days. The *Drosophila* were stocked 30 per vial, and each vial contained a filter paper saturated with 300 μl of 5% H_2_O_2_ in 5% sucrose. Dead flies were counted every 2 h until all flies were dead.

### Triglyceride Measurements

The measurements followed the protocol of a serum triglyceride determination kit (Sigma-Aldrich, USA), a BCA protein assay kit (Thermo Scientific Pierce, USA), and the previous paper [7]. The flies were fed SM+CPE or SM for 2 weeks. Ten flies were homogenized using 100 μl of 1x phosphate-buffered saline (Welgene) containing 0.5% Tween-20 (Tris‐buffered saline with Tween‐20; Biosesang, Korea) and incubated at 70°C for 10 min. After that, they were centrifuged for 3 min, and 20 μl of supernatant was incubated with triglyceride reagent for 1 h at 37°C and detected at OD545 nm using a fluorescence spectrometer (SpectraMax M2, Molecular Devices, USA). The triglyceride level was normalized to protein amounts in each homogenate.

### Statistics

Statistical analysis was performed using SPSS 20 (IBM Corporation, USA). Lifespan data were analyzed by Kaplan-Meier log-rank tests. All other measurements were analyzed by *t*-test. Differences were considered significant when *p* < 0.005.

## Results and Discussion

### Effect of *Crataegus pinnatifida* Extract (CPE) on Lifespan in Flies

**Fig. 1:**
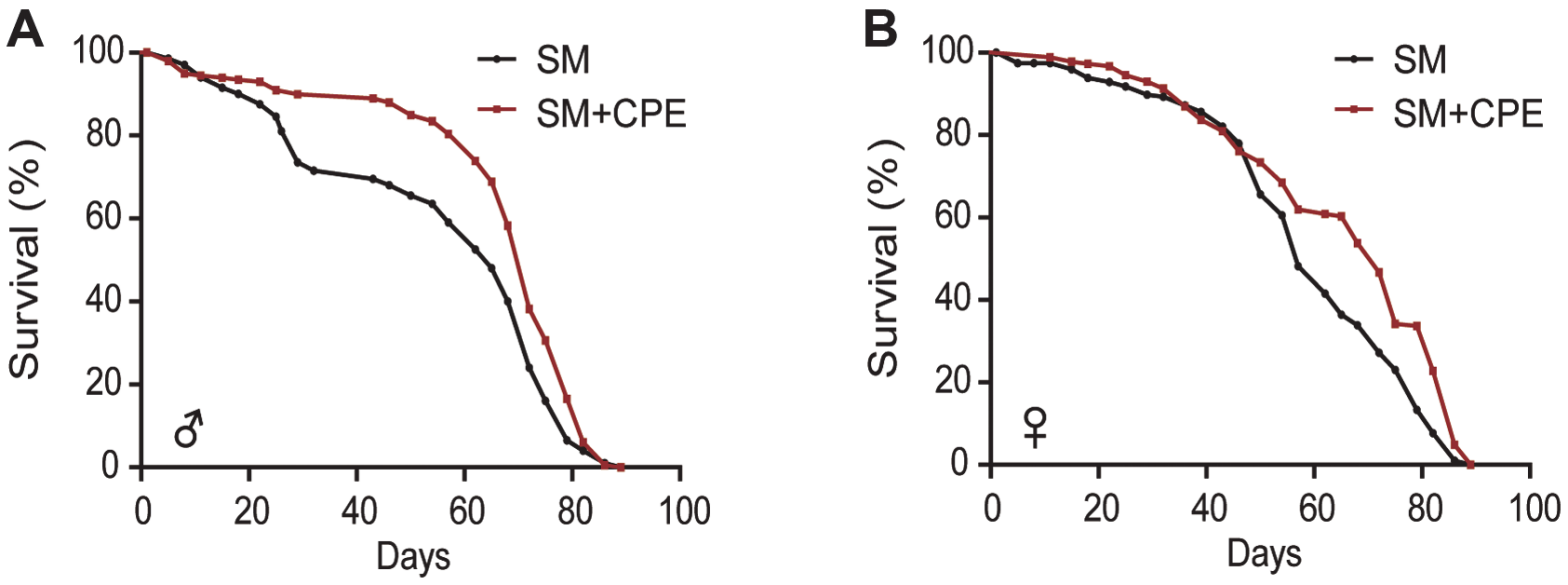
Effect of *C. pinnatifida* extract (CPE) on lifespan in *D. melanogaster*. (**A**) Male (**B**) female lifespan curves of *D. melanogaster* (*n* = 200) fed SM+CPE(1 mg/ml) or SM. Data were recorded until the last *Drosophila* died. The p values are based on Kaplan-Meier test of spss. (*p* < 0.0001; both male and female).

The correlation between CPE and lifespan was examined, and CPE at a concentration of 1 mg/ml was found to significantly prolong the lifespan of *Drosophila*. In males, the median lifespan was 72 days for the SM+CPE group and 65 days for the SM group. In females, the median lifespan was 72 days for the SM+CPE group compared to 57 days for the SM group. In other words, CPE ingestion increased *Drosophila* lifespan regardless of sex (Figs. 1A and 1B). Only the change in the median lifespan could be confirmed without changing the maximum lifespan. Similar results were found in previous studies that confirmed the longevity effect of blueberries [8], black tea [9], and nectarine [10] in *Drosophila*.

### Effect of CPE on Aging-Related Genes

**Fig. 2:**
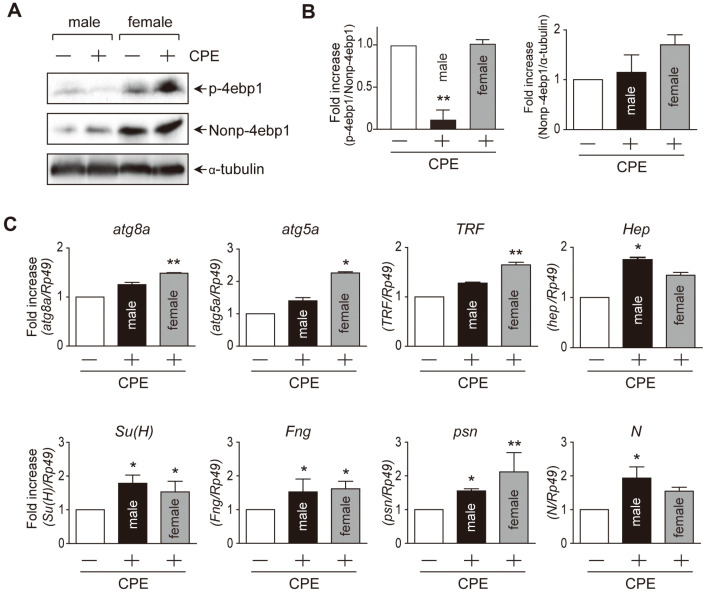
Effect of CPE on aging-related genes. Flies were maintained on standard medium or CPE supplemented on standard medium for a month. (**A**) Western blot analysis using nonp-4EBP. (**B**) Quantification data of (**A**) results. (**C**) The effect of CPE on mRNA of 8 aging related genes compared with SM feeding group. Data were expressed as mean ± SD.

Furthermore, we identified the expression levels of longevity-related genes in fruit flies with or without CPE for one month. Western blotting was performed using 4E-BP to determine the changes in mTOR mechanism, which is a representative mechanism of aging. The expression and phosphorylation levels of 4E-BP were consistent with those of previous studies after treatment with CPE (Figs. 2A and 2B). The results showed that the phosphorylation of 4E-BP decreased more dramatically in males [11]. Autophagy, which is affected by mTOR, is an evolutionarily conserved catabolism that plays an essential role in homeostasis and is a representative mechanism of longevity regulation. To confirm whether CPE intake affects autophagy, qPCR was used to determine the expression levels of *Atg5* and *Atg8a*, which are the two genes involved in membrane expansion and completion. The results showed that CPE significantly affected autophagy in female fruit flies (Fig. 2C). Mutations in the *Atg8a* gene in *Drosophila* resulted in a reduced lifespan, whereas overexpression increased the lifespan by 56% [12]. Overexpression of *Atg5* in mice activates autophagy and increases life expectancy by 17% [13]. That is, autophagy affects lifespan, and it has been affected by CPE intake. In addition, according to previous studies, inhibition of rapamycin-induced mTOR pathways can increase the lifespan of mice, with more sex-dependent responses in women [14]. In *Drosophila*, rapamycin has a greater lifespan effect on females [15]. In this study, the degree of increase in the lifespan of females was higher than that of males, and the expression levels of 4E-BP, *Atg8a*, and *Atg5*, which were affected by the mTOR mechanism, were greater in females than in males. This may be the cause of sexual dimorphism during the aging process [16].

**Fig. 3:**
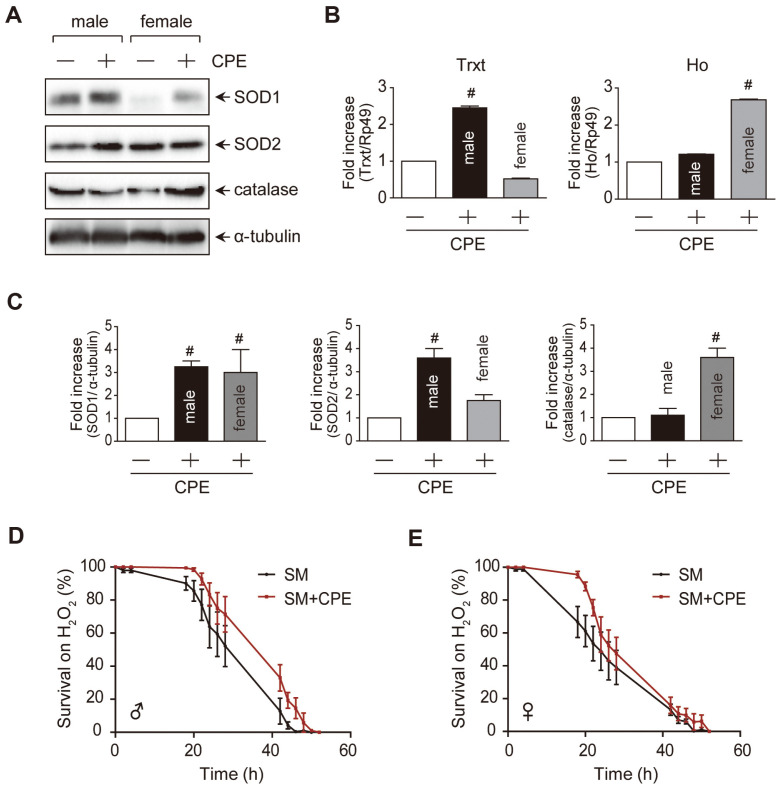
Effect of CPE on oxidative stress. Flies were maintained on standard medium or CPE supplemented on standard medium. In (**A**), (**B**) and (**C**) graph, *w^1118^* were fed SM+CPE or SM for a month. In (**D**) and (**E**), flies were fed SM+CPE or SM for 2 weeks. (**A**) Western blot analysis using SOD1, SOD2 and catalase. (**B**) The effect of CPE on mRNA of *Trxt* and *Ho* genes. (**C**) Quantification data of (**A**) results. (**D**) Lifespan curve of male flies exposed to H_2_O_2_. (**E**) Lifespan curve of female flies exposed to H_2_O_2_. Data expressed as mean ± SD. **; *p* < 0.01.

Notch signaling and telomeres are involved in stem cell generation. Notch signaling affects neural stem cell (NSC) regulation, central and peripheral nervous system development, and muscle differentiation [17]. The expression levels of genes involved in each step of Notch signaling were identified using qPCR. *Su(H)*, *Fng*, *Psn*, and *N* were significantly increased in males treated with CPE, whereas Su(H), Fng, and Psn were significantly upregulated in females. TRF is a telomerase inhibitor that functions as a telomere length reporter and affects stem cell production and homeostasis [18]; in particular, telomeres are a hallmark of aging [19]. CPE uptake was found to affect Notch signaling and TRF expression (Fig. 2C). Hep, a gene involved in the stress signaling pathway, was found to increase significantly in males treated with CPE, as confirmed by qPCR (Fig. 3C).

These mechanisms are affected by their interaction with one another. When the mTOR signal is suppressed, translation is degraded and autophagy is activated. ROS can also activate hypophosphorylated 4E-BP by activating Hep and JNK signaling, and therefore, inhibition of translation affects lifespan [20, 21]. This study could identify trends consistent with previous studies showing increased 4E-BP expression, increased autophagy-related gene expression, and increased Hep expression.

### Effect of CPE on Oxidative Stress

The ingestion of CPE not only affected genes involved in aging-related mechanisms but also genes involved in oxidative stress. The expression levels of SOD1, SOD2, and catalase, which are representative antioxidants, were determined by western blotting (Figs. 3A and 3C). In the case of SOD1, both gendered fruit flies showed a significant increase in gene expression. SOD2 expression increased significantly only in males, whereas catalase expression increased significantly only in females. In other words, after ingesting CPE, antioxidant capacity increased significantly in both male and female fruit flies. The effects of antioxidant enzymes on longevity have been demonstrated in several studies. As a result of measuring the lifespan of SOD1 RNAi flies, the median and maximum lifespans were found to be significantly reduced compared to control flies [10]. Similarly, the lifespans of SOD1 mutant yeast and SOD2 mutant yeast were also decreased compared to those of the control [22]. Flies fed with apple, blueberry, and cranberry extracts were found to have significantly increased lifespans, with increased expression levels of antioxidant enzymes [5, 8, 23]. Heme oxygenase also plays a role in immune regulation and prevention of oxidative stress [24]. HO-1 plays an important role in improving vascular function, weakening adipocyte differentiation, and reducing ROS production. It is also translated into the mitochondria, which increases mitochondrial production [25]. Thioredoxin T(TrxT) is a selenoenzyme in the glutathione-glutaredoxin system (NADPH, glutathione reductase, GSH, and Grx) [26]. This enzymatically reducing agent protects against oxidative stress and apoptosis. Thioredoxin interacts with thioredoxin reductase, consumes NADPH, and is effective in reducing disulfide compounds, such as peroxiredoxins and glutathione disulfide [27]. Overexpression of Trxt in *Drosophila* may help prolong lifespan [28, 29]. The Trxt and Ho expression levels were measured (Fig. 3B). In the group fed CPE, Trxt was significantly upregulated in male fruit flies, while Ho expression was significantly increased in female fruit flies. Specifically, the levels of antioxidant enzymes were altered in chronic oxidative stress (Figs. 3B, and 3C). The effect of acute oxidative stress on the lifespan of *Drosophila* fed a diet with or without CPE and hydrogen peroxide was examined. The lifespan was significantly increased in the CPE group, regardless of sex (Figs. 3D and 3E). Fruit flies fed with broccoli extract experience an increased lifespan as well as an increase in the expression of antioxidant genes after treatment with paraquat and H_2_O_2_ [30]. In other words, CPE treatment affected both acute and chronic oxidative stress.

### CPE Does Not Affect Ffecundity, TAG Levels, Locomotion, and Feeding Amount

**Fig. 4:**
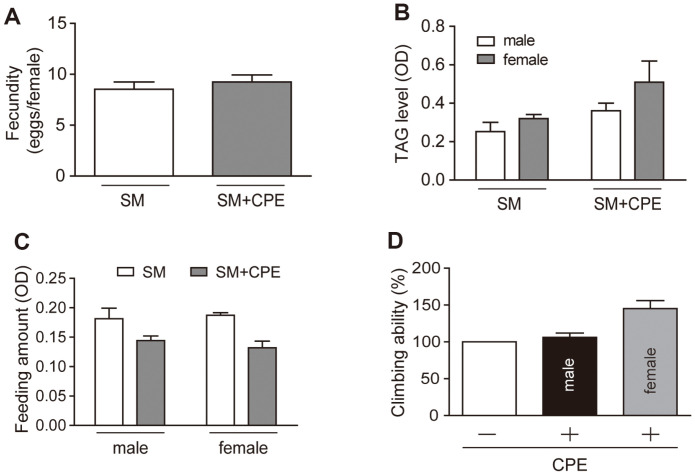
Average of fecundity, TAG level, feeding amount and climbing ability. *w^1118^* of (**B**), (**C**) and (**D**) were maintained on standard medium or CPE supplemented on standard medium for 2 weeks. (**A**) When using 3-day-old *Drosophila* when ingesting SM or SM+CPE to see if there is a difference in intake. (**B**) To confirm TAG level, after flies were homogenized, it was measured at OD 545nm. (**C**) During 10 days, every 24 h, embryos were counted in 10 vials each containing 1 female fly and 2 male flies. (**D**) Vials were tapped to make flies go to the bottom and then the time required for flies to climb up 8 cm was measured. In this figure, there is no significant difference in the data.

As aging progresses, fecundity, motor ability, and metabolism gradually decrease. Therefore, we investigated whether CPE intake affects the deterioration of physical function that occurs as a result of aging. Lifespan increases under diet restriction conditions and so we examined whether CPE affected the amount of feed ingested by fruit flies. No significant differences were found in the feeding amount between the SM and SM+CPE groups, regardless of sex (Fig. 4A). Previous studies have shown that fecundity decreases and TAG levels increase when rapamycin is used [15]. Fecundity and lifespan are mutually exclusive and both are affected by essential amino acids [31, 32]. The removal of germ cells from *C. elegans* increases its lifespan, suggesting that fertility is closely related to aging [33]. Triglycerides are stored in the fat bodies of fruit flies and are the main source of energy. Dietary restriction increases fat storage and triglyceride synthesis and breakdown [34]. The results of this study showed that TAG levels and fecundity did not change significantly after CPE intake (Figs. 4B and 4C).

Finally, muscle loss and decreased muscle function are representative features of aging [35]. In our study, the exercise ability of the CPE-treated group was similar to that of the control group (Fig. 4D), indicating that CPE administration did not impair athletic ability.

These findings confirm that CPE ingestion is useful for increasing both the length and quality of lifespan, regardless of sex. Although functional foods for directly expanding lifespan have not been commercialized until now, much interest in longevity could make them industrial. As mentioned previously, the antioxidant effect has a significant impact on longevity. Because CPE has demonstrated powerful antioxidant and anti-aging effects, it could be considered for commercialization as a potential therapeutic agent for both promoting a healthy lifespan and providing antioxidant benefits.

## Figures and Tables

**Table 1:** The oligomer sequences used in the PCR reaction.

Genes	Forward (5’- 3’)	Reverse (5’- 3’)
*Trxt*	AGAGCCCTGATTGTATTGGC	AGGTCAGATTCGACCACCTC
*Ho*	ATGTCAGCGAGCGAAGAAACAATA	GTACAGTTCGTAGAAGGCCAGGAG
*Atg8a*	ATCGTCGAGAAGGCTCCCAA	TACAGGGAGCCCATGGTAGC
*Atg5*	TCAGTTCTGGGCCGTCAATC	CCATTGCAGTTGGGTCTCC
*TRF*	AGTTTCACTTTAAAGTCGCGGACG	TTCGCATGATGACGCCACGAAA
*Hep*	ATGTCCACCATTGAGTTCGAAACG	TATGCTGATACCCGATCCGGAA
*Su(H)*	AACAACAAATGCAGATGTCCTTGC	AGAATCGCTTCTCATTGCCATAGG
*Fng*	TCTCATCAACACGAAATGCTCACA	CTATACTCGTCCAGCAGTTTGACC
*Psn*	TTCGTTATATGGAAGCCCTGAACG	GGGATTCCGAGTCCTCATCTTCTA
*N*	CCATCAGACGGGCATTCAAATCTA	GATGCCCCTAACACTGTGTATCTG
*Rp49*	CCAAGATCGTGAAGAAGCG	TGGGCATCAGATACTGTC
